# Supplementary material for: An open-source framework for large-scale, flexible evaluation of biomedical text mining systems
Source: J Biomed Discov Collab. 2008 Jan 29;3:1. doi: 10.1186/1747-5333-3-1 (PMC2276192; doi:10.1186/1747-5333-3-1)
Supplement: Additional file 1 — Evaluation platform source code. The additional file contains the software evaluation framework discussed in this paper. The most current release can be downloaded from SourceForge.net [1]. See the accompanying README file for instructions on installation and use. Also included in the distribution is a collection of UIMA wrappers for some commonly used BioNLP tools and annotated corpora. [file 1747-5333-3-1-S1.zip › bionlp_uima_repository-1.0.1/docs/api/src-html/edu/uchsc/ccp/util/nlp/mention/SlotMentionTypes.html]

```
001    package edu.uchsc.ccp.util.nlp.mention;
002    
003    public interface SlotMentionTypes {
004        /** SlotMention Identifier - if this changes, Annotation DB must be adjusted accordingly */
005        public static final String SLOT_MENTION_TYPE = "slot mention";
006        //public static final String SLOT_VALUE_TYPE = "slot value";
007    
008        /** Document-level Slot Mentions */
009        public static final String PROCESSED_TEXT_SLOT = "processed text";
010        
011        /** Syntactic Slot Mentions */
012        public static final String TOKEN_PARTOFSPEECH = "partOfSpeech";
013        public static final String TOKEN_LEMMA = "lemma";
014        public static final String TOKEN_STEM = "stem";
015        public static final String TOKEN_NUMBER = "tokenNumber";
016        public static final String TOKEN_IS_ANAPHOR_FOR = "isAnaphorFor";
017        
018        public static final String TOKEN_TYPED_DEPENDENCY = "typedDependency";
019    //    public static final String TYPEDEPENDENCY_GOVERNOR_TOKEN = "governorToken";
020    //    public static final String TYPEDEPENDENCY_DEPENDENT_TOKEN = "dependentToken";
021    //    public static final String TYPEDEPENDENCY_RELATION = "relation";
022        
023        public static final String PHRASE_TYPE = "phraseType";
024        public static final String PHRASE_LEVEL = "phraseLevel";
025        public static final String PHRASE_NUMBER = "phraseNumber";
026        
027        public static final String CLAUSE_TYPE = "clauseType";
028        public static final String CLAUSE_LEVEL = "clauseLevel";
029        public static final String CLAUSE_NUMBER = "clauseNumber";
030        
031        /** Semantic Slot Mentions */
032        public static final String PROTEIN_ENTREZGENEID = "entrez_gene_id";
033        public static final String EXPRESSION_LOCATION = "location";
034        public static final String EXPRESSION_EXPRESSED_GENE_OR_GENE_PRODUCT = "expressed gene or gene product";
035        
036        public static final String PART_OF = "part of";
037        
038        /** for Mutation project */
039        public static final String BIOLOGICAL_SEQUENCE_SEQUENCE = "sequence";
040        
041        /** Document Section and SubSection slots */
042        public static final String DOCUMENT_SECTION_NAME = "section name";
043        public static final String DOCUMENT_SUBSECTION_NAME = "subsection name";
044        public static final String TABLE_ID = "table id";
045        public static final String FIGURE_ID = "figure id";
046        
047        
048        /** for Olga's concept recognizers */
049        public static final String MESH_ID = "MeshID";
050        
051        /** for MedLEE */
052        public static final String MEDLEE_ID = "MedLEE ID";
053        
054        /** For Species Classification */
055        public static final String NCBI_TAX_ID = "NCBI Taxon ID";
056        
057    }
```
